# The complete chloroplast genome of *Geodorum eulophioides* (Orchidaceae)

**DOI:** 10.1080/23802359.2020.1778558

**Published:** 2020-06-17

**Authors:** Xin-Yi Wu, Jian-Bing Chen, Zhi-Cong Huang, Wei-Rong Liu, Ting-Zhang Li

**Affiliations:** a Key Laboratory of National Forestry and Grassland Administration for Orchid Conservation and Utilization, Shenzhen, China; b Shenzhen Key Laboratory for Orchid Conservation and Utilization, The National Orchid Conservation Center of China and The Orchid Conservation and Research Center of Shenzhen, Shenzhen, China

**Keywords:** *Geodorum eulophioides*, chloroplast genome, phylogenetic, illumina sequencing

## Abstract

*Geodorum eulophioides* Schltr., is a critically Endangered orchid (IUCN). In this study, we report the first complete chloroplast (cp) genome of *G. eulophioides* to provide the underlying information for genetic breeding and conservation studies of this species. The cp genome sequence of *G. eulophioides* is 149,466 bp in length, which contains one large single-copy region (LSC, 85,436 bp), one small single-copy region (SSC, 14,086 bp), and two inverted repeat regions (IRs, 24,972 bp). The cp genome encoded 177 genes, of which 106 were unique genes (78 protein-coding genes, 24 tRNAs, and 4 rRNAs). Phylogenetic analysis showed that *G. eulophioides* is closely related to the genera *Eulophia*.

*Geodorum* Jacks (Orchidaceae) has approximately 10 species that are distributed from tropical Asia to Australia and the South-West Pacific Islands. There are six species in China, including two endemic species (Chen et al. 2009).

*Geodorum eulophioides* Schltr. (1921) is a critically Endangered orchid (IUCN), which grows along the valley with an elevation of 800 meters. It has a few known populations: two found in southwest China (Liu 2010) and a location in central Myanmar (Tanaka et al. 2011). In this study, we sequenced the complete plastome of *G. eulophioides* to provide the underlying information for genetic breeding and conservation studies of this species.

Leaf samples of *G. eulophioides* were obtained from the Orchid Conservation and Research Center of Shenzhen and specimens that were deposited in the China National Orchid Conservation Center Herbarium (NOCC; specimen code Z.J. Liu 8824). Total genomic DNA was extracted from fresh leaves using the modified CTAB procedure method (Doyle and Doyle 1987) and sequenced by using Illumina Hiseq 4000 platform (San Diego, CA, USA). After assembled by MITObim v1.8 (Hahn et al. 2013), the obtained scaffolds and contigs were annotated with CpGAVAS (Liu et al. 2012), finally adjusted by Geneious version 11.1.15 (Kearse et al. 2012). This newly obtained complete cp genome sequence was submitted to GenBank (ID:MK848065).

The cp genome sequence of *G. eulophioides* is 149,466 bp in length, which contains one large single-copy region (LSC, 85,436 bp), one small single-copy region (SSC, 14,086 bp), and two inverted repeat regions (IRs, 24,972 bp). The cp genome encoded 177 genes, of which 106 were unique genes (78 protein-coding genes, 24 tRNAs, and 4 rRNAs).

To further investigate its phylogenetic position, we used RAxML-HPC2 on XSEDE 8.2.12 (Stamatakis et al. 2008) to construct a maximum likelihood tree with other 13 published cp genomes of Orchidaceae. We computed the branch support with 1000 bootstrap replicates by Stamatakis et al. (2008). Phylogenetic analysis showed that *G. eulophioides* is closely related to the genera *Eulophia* (Figure 1). The determination of the complete plastid genome sequences provided new molecular data, which is helpful to illuminate evolution mechanism in Orchidaceae.

**Figure 1.**
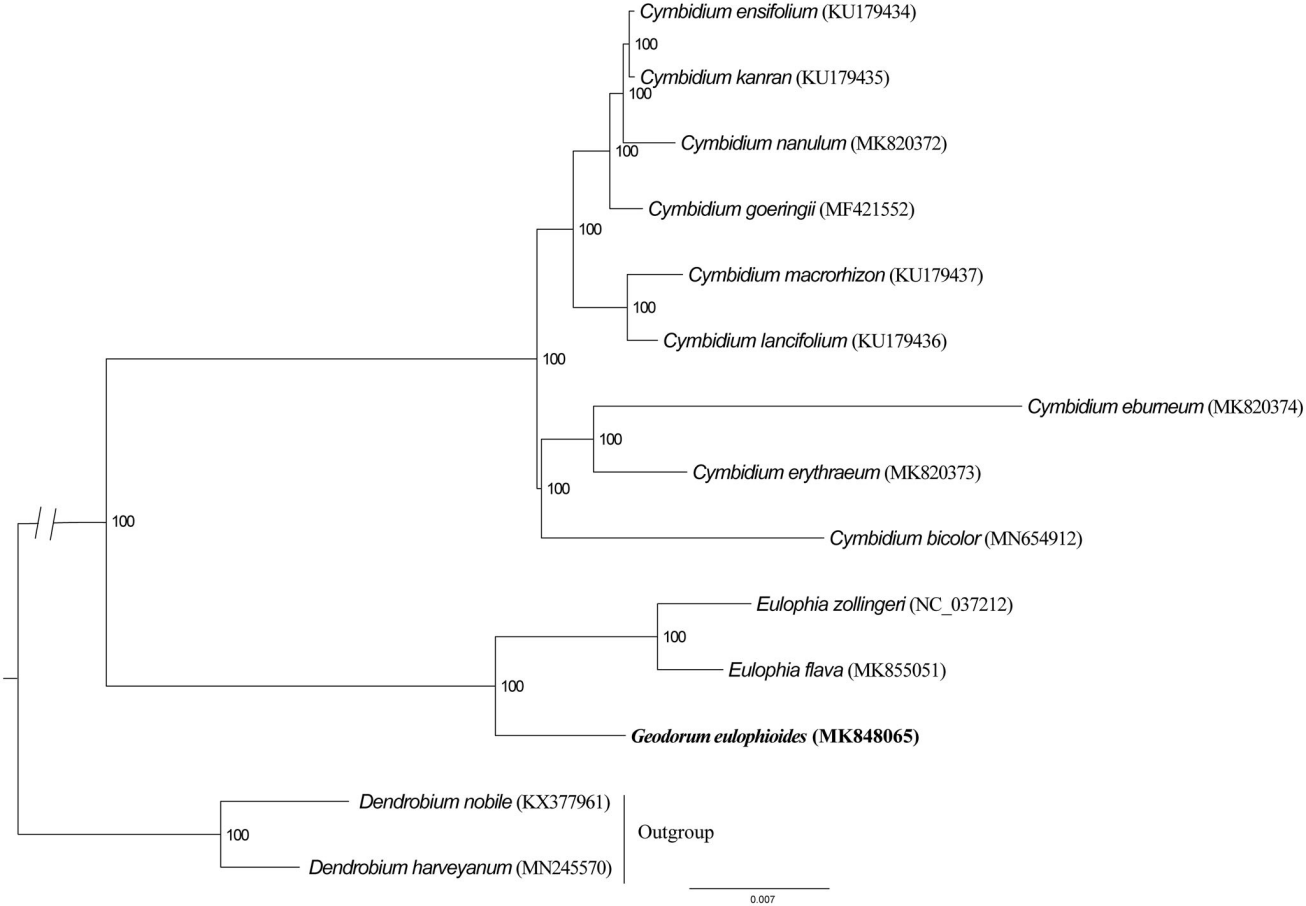
Phylogenetic position of *Geodorum eulophioides* inferred by maximum likelihood (ML) of complete cp genome. The bootstrap values are shown next to the nodes.

## Data Availability

The data that support the findings of this study are openly available in GenBank of NCBI at https://www.ncbi.nlm.nih.gov/, reference number [MK848065].
